# Rock Physical Controls on Production-induced Compaction in the Groningen Field

**DOI:** 10.1038/s41598-018-25455-z

**Published:** 2018-05-08

**Authors:** Sander Hol, Arjan van der Linden, Stijn Bierman, Fons Marcelis, Axel Makurat

**Affiliations:** 1 Shell Global Solutions International B.V., Rock & Fluid Science, Kessler Park 1, 2288 GS Rijswijk, The Netherlands; 2 Shell Global Solutions International B.V., Statistics & Data Science, Grasweg 31, 1031 HW Amsterdam, The Netherlands

## Abstract

Advancing production from the Groningen gas field to full depletion generates substantial, field-scale deformation, and surface subsidence. Quantifying associated risk requires understanding physical processes in the subsurface, in particular those related to deformation of the Permian sandstone reservoir. Here, we report the results of a large experimental study, using fresh core material taken from the center of the field. By subjecting the material to depletion and slight unloading, complemented with a range of rock property measurements, we determine what rock physical properties control production-induced compaction in the material. Our results show that, although a large part of the deformation can be explained by classical linear poroelasticity, the contribution of inelastic (permanent) deformation is also significant. In fact, it increases with progressing pressure depletion, i.e. with increasing production. Utilizing univariate and multivariate statistical methods, we explain the additional inelastic deformation by direct effects of porosity, packing, and mineral composition. These proxies are in turn related to the depositional setting of the Permian reservoir. Our findings suggest that field-scale subsidence may not only be related to the often-used rock porosity, but also to packing, and composition, hence the local depositional environment. This motivates alternative assessments of human-induced mechanical effects in sedimentary systems.

## Introduction

Induced seismicity and surface subsidence are occurring worldwide, with increased frequency, and are associated to a number of different anthropogenic activities^1,2^. In the case of the Groningen gas field, The Netherlands, advancing gas production has generated substantial field-scale deformation, expressed as local seismicity and surface subsidence. Part of the deformation is originating from compaction of a well-sorted fluvial-aeolian sandstone reservoir. As public concern grows, fundamental questions about the causes of these effects arise, which in turn affect gas production limits. To this end, it is key to advance our understanding of the physical processes underlying reservoir compaction, and the mechanisms of fault slip. History-matching and prediction of subsidence is usually performed using analytical and numerical models based on the Geertsma equation^3^ that considers the stress-strain response of an elastic half-space inclusion (cf.^4^).

In this paper, we report new rock physical controls on compaction, obtained from a geomechanical laboratory study using selected samples from Permian reservoir sandstone obtained from a research well in Zeerijp, drilled in 2015 by operator Nederlandse Aardolie Maatschappij (NAM). The work focuses specifically on the magnitude and mechanisms of reservoir compaction, and relates these to a range of relevant boundary and imposed conditions that control the geomechanical state of the reservoir sandstone. Depletion-induced reservoir compaction is expected to be controlled by various grain-scale physical mechanisms that include (sub-)critical cracking or particle re-arrangement^5,6^, as well as intergranular pressure solution creep^7,8^. Aside from identifying the most important grain-scale mechanisms that act on both the short and the long term, also the spatial and intra-reservoir nature of these mechanisms is important for understanding and predicting the relation between production, reservoir-scale deformation, and surface effects. The experimental work reported here was therefore carried out in the most detailed manner possible, and performed in large quantities, allowing to investigate the rock physical controls on the compaction behavior in combination with statistical modeling. Specifically, we report new relationships between stress and strain response during pore pressure depletion testing, with particle packing properties from laser particle size analysis, and composition from X-Ray Diffraction (XRD) techniques. In doing this, we systematically separate permanent and non-permanent deformation.

## Stress-strain behavior and magnitude-controlling rock properties

The best available experimental protocol for characterizing the mechanical response of reservoir sandstone subjected to gas production is to apply the Pore Pressure Depletion (PPD) protocol^9^. More than 40 PPD experiments were conducted, with a total equivalent time-burden of approximately 10 years testing time, closely following and expanding on the International Society of Rock Mechanics (ISRM) Suggested Method for PPD testing under uniaxial-strain boundary conditions^9^, i.e. using the Uniaxial-strain PPD (UPPD) protocol. Most tests were executed by subjecting a cylindrical rock sample to three depletion steps that included a number of unloading-reloading steps to resolve the contribution of elastic strain to the total strain per depletion step. Data are compared with a) particle packing properties derived from particle size distribution data, b) composition derived from X-Ray Diffraction (XRD) analyses, as well as c) porosity and permeability, to determine the main controlling factors that affect rock compaction behavior.

**Figure 1:**
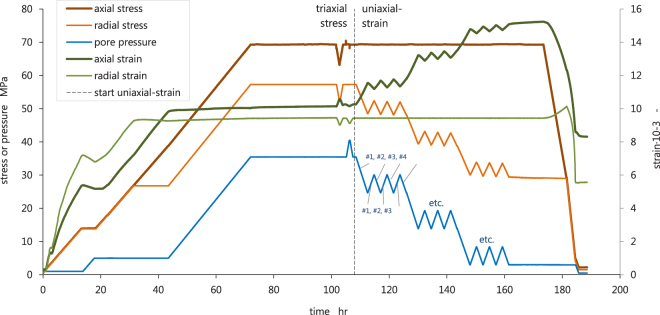
Axial stress, radial stress, pore pressure (left axis), and axial and radial strains (right axis), versus time, plotted for a complete test following the UPPD/M protocol. Sample ZRP-3A_123D is here first loaded to *in-situ* stress and pore pressure conditions (0–70 hr), then stabilized (70–105 hr), and finally depleted under uniaxial-strain boundary conditions (105–180 hr, with zero change in radial strain). The core of the UPPD/M protocol consists of three pore pressure steps (35 MPa to 25 MPa, 25 MPa to 15 MPa, and 15 MPa to 3 MPa) that are each followed by three inflation-deflation steps (note the spikes in pore pressure, radial stress, and responding axial strain). All inflation and deflation steps were sequentially numbered, e.g. the inflation steps number from #1 to #9, whereby steps #3, #6, and #9 were parameterized as purely elastic response. The tangent of the strain development with pore pressure change is taken as the uniaxial-strain compressibility C_m_, either as total C_m_, or elastic C_m_, depending on the loading direction. The inelastic uniaxial-strain compressibility C_m_ is then obtained by subtracting the elastic C_m_ from the total C_m_ per pore pressure step.

**Figure 2:**
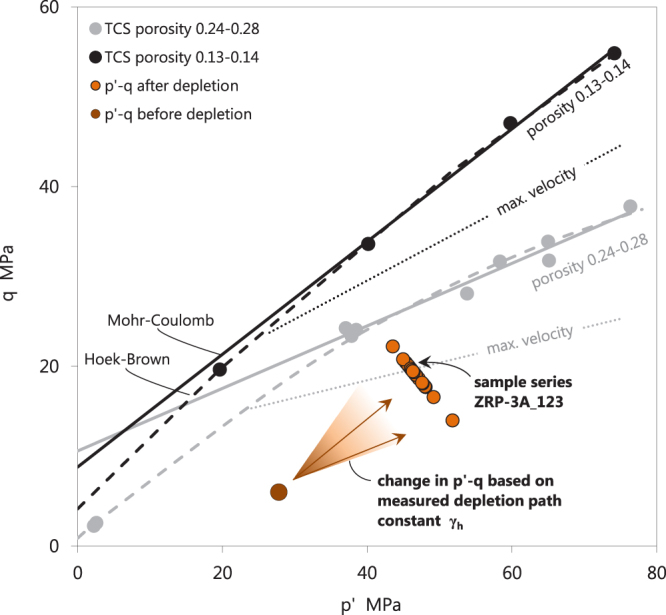
Overview in p′-q stress space of the Triaxial Compressive Strength (TCS) and Pore Pressure Depletion (PPD) tests performed using Zeerijp sandstone samples. The TCS tests were performed using samples with an end-member porosity, and are presented in black (low porosity 0.13–0.14) and grey (high porosity 0.24–0.28). Solid points represent the peak stress at failure, for which per sample the Mohr-Coulomb (solid line) and Hoek-Brown (dashed line) criteria have been fit. A fit of the maximum ultrasonic p-wave velocities during axial loading is shown in dotted lines, for both data sets, to indicate the likely location of the critical state line. The dotted line physically indicates the boundary between densification-dominated deformation (below), and cracking-dominated deformation (zone between maximum velocity and failure lines). The PPD tests start at the virgin *in-situ* stress (dark brown point), and by the responding radial stress change follow a stress path with a fixed depletion path constant γ_h_ = ΔS_rad_/ΔP_p_. Final p′-q conditions for all PPD tests (light brown points) are hence at higher p′-q, but still below the failure line and within the brittle field. No evidence for shear or compactant collapse was observed. However, note that the p′-q at post-depletion conditions for sample series ZRP-3A_123 with a porosity of 0.22 are approaching the maximum velocity line, and may hence be close to the critical state line when fully depleted. Refer here to the stress path in Fig. 1, and the acoustic emissions data in Fig. 6, for sample ZRP-3A_123D specifically.

A relationship between the total uniaxial-strain compressibility C_m_ and porosity is reported extensively in the industry literature for sandstone from the Groningen Field, and North Sea Fields^10–15^, employing not only the pore pressure depletion protocol, but also oedometric loading techniques. However, our testing methods not only allowed for the determination of total C_m_, but also the elastic C_m_ (refer to axial strain, pore pressure, and radial stress, plotted in Fig. 1 between 105 hr and 160 hr). Parameterizing the stress-strain response during the final deflation step following each first depletion step yields the recoverable (elastic) response for that particular pore pressure step, and the stress response required to maintain the uniaxial-strain boundary conditions from which the horizontal depletion path constant γ_h_ can be calculated using the expression γ_h_ = ΔS_rad_/ΔP_p_^16^, where ΔS_rad_ is the change in radial stress applied by the confining pressure, and ΔP_p_ is the change in pore pressure, as recorded during the execution of the UPPD protocol. Basic strength characterization by means of Triaxial Compressive Strength (TCS) testing is presented in Fig. 2 in p′-q space, presenting the mean effective stress p′ = ½(S_ax_′ + S_rad_′) and deviator stress q = ½(S_ax_ − S_rad_) of selected sample sets, where S_ax_ is the applied axial stress, from which we infer that the failure behavior displays a classic brittle shear response. Clearly, low porosity samples have a higher friction angle (slope of the linear fit). Brittle shear was confirmed by visual inspection of the samples that all showed distinct failure planes. The stress path that was followed by the samples subjected to pore pressure depletion did not intersect the failure line. None of the experimentally depleted samples failed catastrophically by localized shear or compaction deformation.

### Elastic (recoverable) uniaxial-strain response

**Figure 3:**
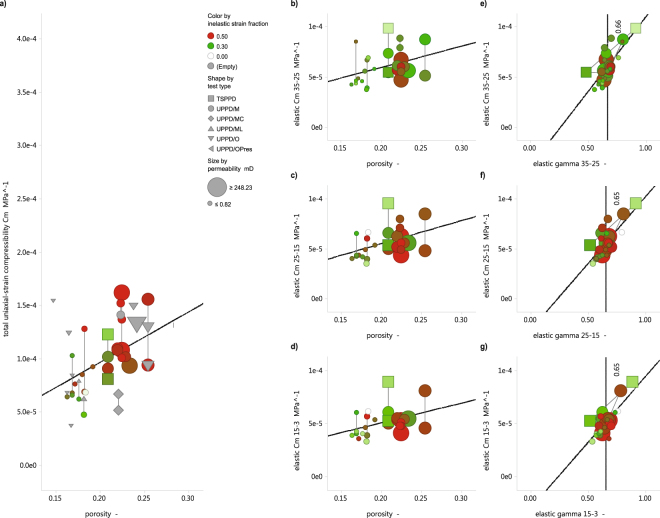
(**a**) Total uniaxial-strain compressibility C_m_ for all tested samples parameterized based on the axial strain and pore pressure change presented in Fig. 1. Shape indicates the various loading protocols employed, with the multicycle Uniaxial-strain Pore Pressure Depletion (UPPD/M) being the most used. Color gradients indicate the fraction of inelastic strain between 0 and 0.3 (white-green), and 0.3–0.5 (gree-red). Note, the clear increase in C_m_ with increasing porosity, as well as the higher permeability and larger fraction of inelastic strain at high C_m_. (**b**–**d**) Uniaxial-strain compressibility C_m_ for each depletion step (pore pressures 35 MPa-25 MPa, 25 MPa-15 MPa, and 15 MPa-3 MPa), as parameterized using the final inflation cycle versus the porosity. Note, the consistent, depletion-independent increasing trend, which suggests that the elastic response in independent of the stress history. (**e**–**g**) Uniaxial-strain compressibility C_m_ for each depletion step versus the measured γ_h_ for all samples. The mean γ_h_ around 0.66, and the data spread, is in line with expected linear poroelastic behavior. Note, that also the TSPPD tests, where a value for γ_h_ was imposed rather than measured, are part of the same trend.

Comparing now, the elastic strain response of the sandstone samples with their compositional and structural properties shows that only porosity is close to significant in predicting strain. The p-values for elastic C_m_ versus porosity relationships are 0.0608 for step 35 MPa to 25 MPa, 0.0225 for step 25 MPa to 15 MPa, and 0.0389 for step 15 MPa to 3 MPa. Evidently, the null hypothesis for the correlation made for the step 35 MPa to 25 MPa cannot be rejected. Although a dependence of the elastic response on the rock properties is absent, a positive relationship between the horizontal depletion path constant γ_h_ and the elastic C_m_ can be observed in Fig. 3e–g. This shows that a constitutive relationship could exist between the axial strain, and radial stress, responses to the re-pressurization of the pore space, i.e. high axial strain is consequently related to large radial stress drop, and vice versa, which is not necessarily dependent on a measured material property.

### Inelastic (permanent) uniaxial-strain response

**Figure 4:**
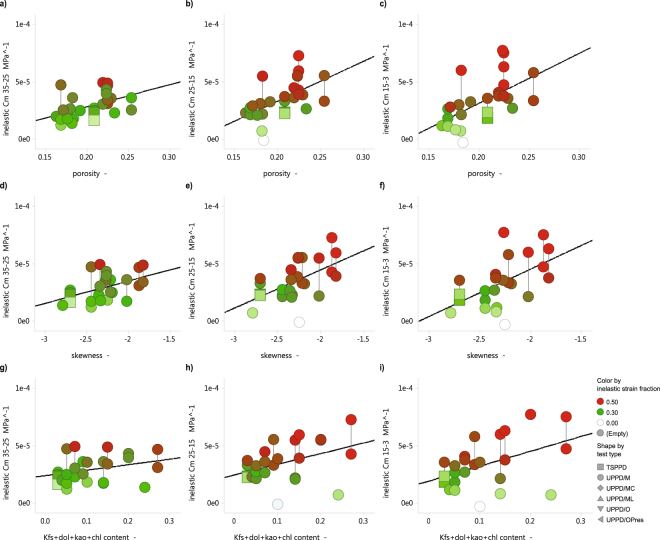
(**a**–**c**) Inelastic uniaxial-strain compressibility C_m_ for each depletion step (pore pressures 35 MPa-25 MPa, 25 MPa-15 MPa, and 15 MPa-3 MPa), calculated by subtracting the total Cm and the elastic Cm, versus porosity. (**d**–**f**) Inelastic Cm for all depletion steps versus skewness of the sandstone particle size distribution. A low value of skewness means a relative abundance of fine particles in the rock particle packing, increasing the coordination number. g)-i) Inelastic Cm for all depletion steps versus the total fraction of “weak” minerals, viz. K-feldspar, dolomite, kaolinite, and chlorite minerals. Note, that the outliers at the bottom of (**h**,**i**) are samples with a porosity lower than 0.19. In all plots shape indicates the various loading protocols employed, with the multicycle Uniaxial-strain Pore Pressure Depletion (UPPD/M) being the most used. Color gradients indicate the fraction of inelastic strain between 0 and 0.3 (white-green), and 0.3–0.5 (gree-red). General observation is that the inelastic Cm increases with depletion, and is related to porosity, skewness, and “weak” mineral content.

Values for the computed inelastic C_m_ range roughly between 1 ∙ 10^−5^ MPa^−1^ and 1∙10^−4^ MPa^−1^ (Fig. 4). Leaching with acid solution of two of the samples resulted in roughly 20% more permanent axial strain, and no difference in radial stress response γ_h_ compared to unleached control samples. Comparing now the inelastic C_m_ (Fig. 4) with the elastic C_m_ (Fig. 3), shows that the inelastic strain ratio (elastic C_m_ over total C_m_) varies from 0.42 for the step 35 MPa-25 MPa, to 0.71, and 0.75, for the steps 25 MPa-15 MPa, and 15 MPa-3 MPa respectively. We performed univariate and multivariate analyses using the inelastic C_m_, and explanatory variables related to packing, mineral composition, and the testing environment. The strongest dependencies were found to be: 1) porosity (Fig. 4a–c), 2) skewness of the particle size distribution (Fig. 4d–f), and 3) “weak” mineral content, in particular when porosity is greater than 0.19 (Fig. 4g–i). A slight effect of temperature was found, notably a non-systematic, slightly lower strain at 60 °C and 100 °C, which is consistent with findings reporting in the literature for this rock at the specific conditions^10^.

First, inelastic C_m_ versus porosity is plotted in Fig. 4a–c for all samples tested. Comparing now the depletion steps 35 MPa-25 MPa, 25 MPa-15 MPa, and 15 MPa-3 MPa (Fig. 4), reveals that the inelastic C_m_ displays a stronger positive dependence on porosity as depletion progresses, and increases its magnitude from approximately 1∙10^−5^ MPa^−1^ at a porosity of 0.15, to 4∙10^−5^ MPa^−1^ to 6∙10^−5^ MPa^−1^ around a porosity of 0.3 depending on the depletion step (Fig. 4a–c).

Second, a comparison of inelastic C_m_ with mean grain size, sorting, kurtosis, and skewness obtained from the fitted log-normal particle size distribution, reveals that samples that are relatively fine-skewed have lower inelastic C_m_ (Fig. 4d–f). Although porosity (the first proxy, refer to Fig. 4a–c) is likely dependent on skewness to some extent, and hence porosity and skewness cannot be considered entirely independent variables, the trend is important in that it demonstrates that the observed compaction (bulk densification) is controlled by the packing properties of the granular rock. The quantitative evidence provided here for the controlling effect of packing on compaction is new to the problem of reservoir compaction in the Groningen Field.

**Figure 5:**
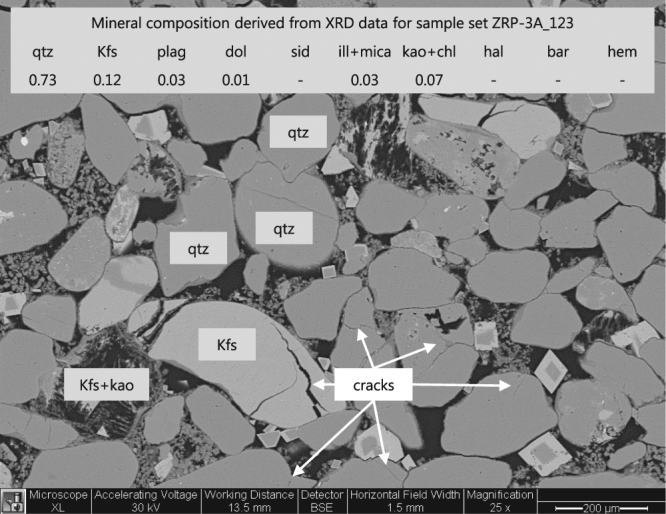
BSEM image of sample ZRP-3A_123BV, showing a representative microstructure of a sandstone sample in the post-testing state with a mean grain size of 140 µm (obtained by Laser particle size analysis). Top part of the image lists the mineral content derived from X-Ray Diffraction analysis using bulk rock material. The image shows multiple examples of leached K-feldspar (grain skeletons), in which also kaolinite is present as reaction product of the leaching product. Quartz minerals contain cracks, as well as grain rimming clay minerals.

Third, based on the geological classification of mineral hardness, the following ranking can be made for the rock-forming minerals in the present samples (strong – weak): quartz – feldspar – dolomite – mica – clays. In principle, we separate hardness for the samples used here based on their mineral hardness contrasts, identifying anything equal or harder than illite as “strong”, Backscattered Scanning Electron Microscopy (BSEM) combined with Electron X-ray Diffraction (EDX) mineral mapping of our samples revealed that the minerals dolomite, and K-feldspar clasts are commonly leached, or cleaved (Fig. 5). We therefore consider those “weak”, and the resulting physical separation of minerals considered here is: “strong” quartz, plagioclase, illite, and mica minerals, versus “weak” K-feldspar, dolomite, kaolinite and chlorite minerals. Comparing now in Fig. 4g–i the inelastic C_m_ with the fraction of weak minerals demonstrates that increasing the volume fraction from 0.05 to 0.3, increases the inelastic C_m_ by from 2∙10^−5^ MPa^−1^ to maximum 8∙10^−5^ MPa^−1^, i.e. by almost a factor of four (Fig. 4g–i). Note here, that the outliers close to zero inelastic strain are all samples with a porosity lower than 0.19, which is similarly observed by Wong and Baud^17^ in carbonates subjected to hydrostatic loading. Comparing now each depletion step (step 35 MPa-25 MPa in Fig. 4g versus step 25 MPa-15 MPa in Fig. 4h versus step 15 MPa-3 MPa in Fig. 4i), reveals that the slope of the linear fits steepens with depletion indicating that the presence of weak minerals in the microstructure appears to control the progression of inelastic strain in the samples. Note also, that extrapolation of the linear fits presented in Fig. 4i, to the hypothetical case of only strong minerals predicts close to zero inelastic C_m_, as the linear fit in Fig. 4i projects through the origin. This shows that the magnitude of the inelastic C_m_ is almost fully controlled by the presence of “weak” minerals in the present samples. In addition to the effect of packing on compaction, the role of mineralogy is also new to the case of reservoir compaction in the Groningen Field. Further to the direct effect of mineralogy, the data also suggest that samples with high porosity, i.e. with a porosity greater than 0.19, display a weak dependence of inelastic C_m_ on kaolinite content in the clay fraction of the ground samples separated for XRD analysis. Close to no dependence on chlorite content was observed. Note, that the samples with high kaolinite content contain relatively more K-feldspar. This suggests that the stability of the samples tested is also affected by the presence of specific clay minerals. A special note with regards to kaolinite is that the clay fraction results might underestimate the kaolinite content since its crystals are relatively coarse and might hence be part of the bulk fraction.

### Acoustic Emission (AE) data

**Figure 6:**
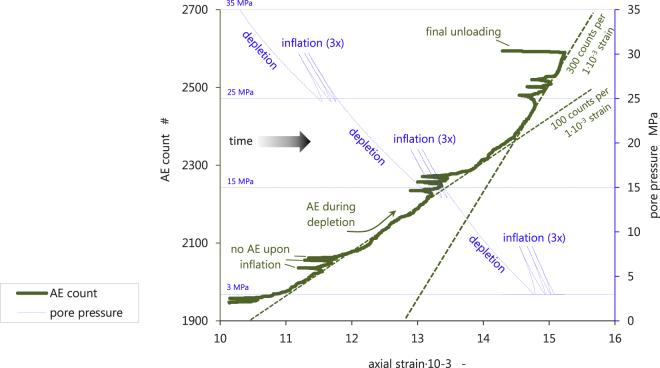
Acoustic Emission (AE) count (left axis) and pore pressure (right axis) versus axial strain for the full depletion trajectory within a UPPD/M protocol. Sample tested is ZRP-3A_123DV. Note, that no AE activity can be seen during inflation-deflation steps, and that a clear kink in the global AE count versus strain trend can be observed around 14.5∙10^−3^ axial strain.

The recording of Acoustic Emission (AE) is generally used to probe cracking activity in materials, and is used in rock deformation studies to identify whether brittle deformation mechanisms are operative (e.g.^18^). AE counting during a UPPD/M test using representative sample material (ZRP-3A_123D with a porosity of 0.22) revealed that the number of AE events increases as depletion progresses (Fig. 6). From the cumulative AE count versus axial strain recorded, two observations can be made. First, comparing the inflation-depletion cycles with the main pore pressure depletion steps, shows that AE events are generated only when pore pressure is decreased (not when increased), and hence only when the effective deviatoric stress is increased. The three horizontal spikes observed in Fig. 6 during unloading show no increase in AE count, and each large pore pressure reduction generated an axial strain increase accompanied by the increase in AE count. Each re-loading step generates close to zero AE count, but AE count increases again after the specific previous load condition has been exceeded (Fig. 6). This suggests that the AE activity is associated with virgin loading only, i.e. it is related to the deformation mechanism responsible for the inelastic strain observed. Second, a slope change in the AE count versus axial strain trend can be observed around 14.5∙10^−3^ strain. In the first part of the experiment, i.e. the depletion steps 35 MPa to 25 MPa, 25 MPa to 15 MPa, and (partly) below 15 MPa, the broad increase in AE count is approximately 100 counts per 1∙10^−3^ strain. The AE rate then suddenly increases around 14.5∙10^−3^ strain to approximately 300 counts per 1∙10^−3^ strain, for which the corresponding pore pressure is close to 10 MPa (Fig. 6). Finally, note that creep during the pore pressure cycle 15 MPa–20 MPa – 15 MPa – results in a deviation from the global AE count versus axial strain trend of 100 counts per 1∙10^−3^ strain.

### Interdependences of variables by means of statistical modeling

Statistical testing was carried out to quantify the relationships between the stress-strain response measured and the physical controls presented in Sections 2.1 and 2.2. As an initial screening test, univariate linear regression models were used to model each of a number of response variables as a function of a number of potential predictor variables (Table 1). At each depth in the core (‘segment’ hereafter), between one and four samples were collected and used for analysis. Some of the measurements are common to all samples taken at a segment, whereas in general variables may be expected to be more similar within segments than they are between segments. To evaluate the ability of the regression models to explain the variability in the response variables, a cross-validation scheme was used in which the regression models were parameterized using training data sets from which (one-at-a-time) data from a segment was omitted (‘test segment’), and the data from this test segment were used to evaluate predictive performance of the models. The Root Mean Squared Error of the Prediction (RMSEP) of the model on the test data was evaluated and used as the main criterion to evaluate the ability of models to explain the variation in the response variables. The RMSEP model was compared to the RMSEPint of models based on the intercept only to compute R^2^ values, which we express as the percentage of the variation explained. The p-values of the slopes of regression variables are also given. These were computed by employing t-tests using the complete data set, i.e. without cross-validation. In Table 1, all available predictor variables with an associated p-value < 0.05 are listed for each response variable, along with their RMSEP and R^2^ values. Some response variables did not have a single predictor variable which was able to explain a statistically significant amount of variation, notably variables E_4–8_#9, E_15-19#4, E_26-30#3, Kb_in.situ_1 (Table 1). However, for most response variables, the predictor variable Porosity_Hg_chloro (sandstone porosity, Table 1) is able to explain a statistically significant amount of variation. For specific response variables, the particle size distribution variables kurtosis, skewness, or sorting, are able to explain a statistically significant amount of variation, and in three cases (nu_4.8_9, Gamma_tot and Gamma_14.3_9). Note however, that when using the cross-validation scheme, models were only able to explain a modest amount of the total variation in the response variables, from a minimum of 6.1% to a maximum of 39.5%. This indicates that one or more of the following could be applicable to the dataset, namely a) there are predictor variables missing from the models, b) the measurement error is relatively large as illustrated by the data spread in Figs 3 and 4, and/or, c) the dependences between variables are in fact of non-linear nature. Finally, univariate testing of the role of mineral content, notably the effect of fraction of the strong minerals quartz, plagioclase, illite and mics, versus the weak minerals K-feldspar, dolomite, kaolinite, and chlorite, is tested specifically for porosity greater than 0.19. This relationship is positive, with a p-value of maximum 0.016.

**Table 1:** Overview of variables tested using univariate statistical models.

Response variable	Predictor Variable	RMSEP_model_	RMSEP_int_	p	R^2^
Cm_inel_15.3	porosity_Hg_Chloro_	1.80E-05	2.10E-05	0.0043	26.5
	skewness_MoM_geo	1.90E-05	2.10E-05	0.0238	18.1
	qtz_bulk + plag_bulk + (ill + mica)_bulk	1.60E-05	2.10E-05	5.00E-04	59.3
	K-f_bulk + dol_bulk + (kao + chl)_bulk	1.50E-05	2.10E-05	5.00E-04	59.7
Cm_inel_25_15	porosity_Hg_Chloro_	1.40E-05	1.70E-05	0.0038	32.2
	skewness_MoM_geo	1.50E-05	1.70E-05	0.0208	22.1
	qtz_bulk + plag_bulk + (ill + mica)_bulk	1.00E-05	1.60E-05	7.00E-04	54.7
	K-f_bulk + dol_bulk + (kao + chl)_bulk	1.00E-05	1.60E-05	5.00E-04	56.0
Cm_inel_35.25	porosity_Hg_Chloro_	1.10E-05	1.20E-05	0.0297	16.0
	skewness_MoM_geo	1.10E-05	1.20E-05	0.039	16.0
	qtz_bulk + plag_bulk + (ill + mica)_bulk	1.00E-05	1.10E-05	0.0160	33.0
	K-f_bulk + dol_bulk + (kao + chl)_bulk	1.00E-05	1.10E-05	0.0117	35.5
Cm_4.8__9	porosity_Hg_Chloro_	1.30E-05	1.40E-05	0.0621	13.8
Cm_14.3__9	porosity_Hg_Chloro_	2.10E-05	2.70E-05	0.0019	39.5
Cm_25.14__5	porosity_Hg_Chloro_	2.20E-05	2.60E-05	0.0027	28.4
Cm_26.30__3	porosity_Hg_Chloro_	1.60E-05	1.70E-05	0.0835	11.4
Cm_35.25__1	porosity_Hg_Chloro_	2.20E-05	2.40E-05	0.0208	16.0
Cm_tot	porosity_Hg_Chloro_	2.50E-05	3.20E-05	<0.001	39.0
E_loading	porosity_Hg_Chloro_	3.076762	3.951904	<0.001	39.4
Gamma_4.8__9	porosity_Hg_Chloro_	0.083072	0.086577	0.1283	7.9
Gamma_14.3__9	phyllosilicate_tot	0.078444	0.081468	0.2257	7.3
	porosity_Hg_Chloro_	0.079615	0.081468	0.2647	4.5
	kurtosis_MoM_geo	0.078471	0.081468	0.2079	7.2
Gamma_15.19__4	porosity_Hg_Chloro_	0.081239	0.091495	0.0221	21.2
Gamma_25.14__5	porosity_Hg_Chloro_	0.077803	0.081876	0.1169	9.7
Gamma_35.25__1	sorting_MoM_geo	0.081081	0.085289	0.1228	9.6
	kurtosis_MoM_geo	0.081178	0.085289	0.1102	9.4
Gamma_tot	phyllosilicate_tot	0.07558	0.078065	0.0981	6.3
	porosity_Hg_Chloro_	0.074479	0.078065	0.0467	9.0
	kurtosis_MoM_geo	0.075647	0.078065	0.0773	6.1
	sorting_MoM_geo	0.075622	0.078065	0.103	6.2
Kb_in.situ__2	porosity_Hg_Chloro_	2.005013	2.37837	4.00E-04	28.9
Kb_loading	porosity_Hg_Chloro_	1.212894	1.448904	1.00E-04	29.9
Ks.loading	sorting_MoM_geo	13.27787	15.06702	3.00E-04	22.3
	kurtosis_MoM_geo	13.47946	15.06702	1.00E-04	20.0
nu_4.8__9	phyllosilicate_tot	0.041394	0.048122	0.0101	26.0
	kurtosis_MoM_geo	0.039269	0.048122	0.0034	33.4
	sorting_MoM_geo	0.044846	0.048122	0.0357	13.2
nu_15.19__4	porosity_Hg_Chloro_	0.041815	0.046297	0.0092	18.4
nu_26.30__3	porosity_Hg_Chloro_	0.038781	0.043406	0.007	20.2
nu_loading	porosity_Hg_Chloro_	0.044462	0.048436	0.0018	15.7

Nomenclature: Cm = uniaxial-strain compressibility, inel = inelastic, el = elastic, prefix “15–3” indicates a pore pressure step, in this case from 15 MPa to 3 MPa pore pressure, Gamma = γ_h_, tot = total averaged over the complete pore pressure depletion step from 35 MPa to 3 MPa, nu = ν, which is the Poisson’s Ratio, MoM = Method of Moments, geo = geometric, Hg = determined using Mercury immersion method, chloro = determined using chloroform immersion method, the prefix “#9” indicates the sequential number of the pore pressure depletion or inflation cycle in the UPPD/M protocol. Minerals are indicated with “qtz” for quartz, “plag” for plagioclase, “dol” for dolomite, “K-f” for K-feldspar, “ill” for illite, “mica” for mica, “kao” for kaolinite, “chl” for chlorite, where the prefix “bulk” refers to the XRD analysis conducted using the bulk crushed fraction.

To investigate whether or not a combination of variables could explain more of the variation, we went on to apply multivariate models. First, we fitted Partial Least Squares (PLS) regression models to each of the response variables using all of the predictor variables (predictor variables were scaled to have zero mean and unit variance). Again, the cross-validation scheme was used to compute the RMSEP for models with increasing numbers of latent variables. Up to five latent variables were included in the PLS models. For none of the predictor variables PLS regression models were found that performed better than simple univariate models in terms of RMSEP. This may be caused by the inclusion of predictor variables which are relatively noisy in the sense that their associated measurement error is relatively large. Since Porosity_Hg_chloro was found to be a useful predictor variable for many of the response variables, we also fitted bivariate regression models with predictor variables additional to Porosity_Hg_chloro. For five of the response variables (Cm_inel_15.3, Cm_inel_25_15, Kb_in.situ_2, Kb_loading and nu_15.19_4), an additional predictor variable was found which explained a statistically significant additional amount of variation in the response variable (refer to Table 2). In all cases, these were particle size distribution variables (sorting, skewness and kurtosis). The bivariate regression models only had a modest improvement in the percentage of variation explained compared to the univariate models, with R^2^ values ranging from 26.7% to 42.7% (Table 2). We note that in this screening test a large number of models have been fitted to the data. The reported results, such as p-values, RMSEP and R^2^ values, may give an over-optimistic view of the strength of evidence of correlations between variables, and the predictive power of models. However, we note that the use of statistical testing of experimental data is uncommon in rock deformation studies, for the simple reason that a great number of potential geological factors and boundary conditions can affect the response measured. From a statistical viewpoint, the reported correlations can therefore be regarded as significant in developing hypotheses for future research.

**Table 2:** Overview of variables tested using bivariate statistical models. Nomenclature similar to Table 1.

Response variable	X1	X2	RMSEP_model_	p (X1)	p (X2)	R^2^
Cm_inel_15.3	porosity_Hg_chloro_	skewness_MoM_geo	1.59E-05	0.0079	0.0416	42.7
Cm_inel_25_15	porosity_Hg_chloro_	skewness_MoM_geo	1.29E-05	0.0074	0.039	42.4
Kb_in.situ_2	porosity_Hg_chloro_	sorting_MoM_geo	1.86E + 00	<0.001	0.0038	38.6
Kb_loading	porosity_Hg_chloro_	sorting_MoM_geo	1.24E + 00	<0.001	0.0374	26.7
nu_15.19_4	porosity_Hg_chloro_	sorting_MoM_geo	3.71E-02	0.0017	0.0325	35.7
		kurtosis_MoM_geo	3.78E-02	0.0069	0.0367	33.5

## Discussion

Modeling subsidence in response to production from the Groningen reservoir is performed to update risk assessments motivated by company internal requirements, and the applicable regulatory framework. One of the most essential parameters in the underlying Geertsma theory is the total uniaxial-strain compressibility C_m_. The total strain in our tests shows relatively little sensitivity to temperature and polar ionic versus nonpolar pore fluids, which is consistent with reports that pressure solution creep is insignificant at the conditions applied^19^. In the case of field-scale models, reservoir porosity as taken from the static reservoir model has long been adopted as the only proxy relevant in the prediction of total C_m_, as consistent with the general experience that the mechanical behavior of poroelastic materials are most sensitive to porosity^20,21^. However, analyzing the laboratory data reported here, by separating the measured depletion strains into elastic and inelastic components, showed a significant contribution of the inelastic (permanent) strain to the total strain. We identified two additional proxies for (inelastic) reservoir compaction, namely a) the skewness of the particle size distribution, and b) the “weak” mineral content, specifically for samples with a porosity greater than 0.19. These proxies are as statistically significant as the reservoir porosity used up to now. Starting with explaining the consistency in elastic behavior of the material, we go on to focus on why such proxies would exist for the inelastic behavior. We thereby aim at understanding controlling deformation mechanisms in the reservoir.

To start, a note on the applicability of our data. The experimental protocol employed here has deliberately not accounted for the difference in reservoir pressure (~10 MPa) during coring versus the reservoir pressure before production (~35 MPa), i.e. all standard depletion experiments commenced at the virgin pore pressure of 35 MPa. Our mechanical result clearly show an effect of this pressure difference. Comparing the three depletion steps employed in our protocol, the steps 25 MPa to 15 MPa, and 15 MPa to 3 MPa, yield higher inelastic strain (Fig. 4), and Acoustic Emissions (AE) count (Fig. 6), compared with the first step from 35 MPa to 25 MPa, which demonstrates that loading of the material in the progressive depletion steps leads to yield. This could be because of loading beyond the pre-loaded state, i.e. the Kaiser effect associated with loading the sample beyond the present-day reservoir pressure, but could also be rapidly accumulating damage associated with passing the critical state line^22,23^ as evidenced by the location of the maximum velocity in p′-q space (Fig. 2).

In the present study, the magnitude of the truly elastic component of the uniaxial-strain compressibility C_m_, named here the “elastic C_m_”, is found to be related to only one material property, namely porosity (Fig. 3), whereby the best correlation exists for the last two depletion steps. However, Fig. 3 shows that the measured elastic responses, namely the axial strain and radial stress change, are correlated in a linear manner (Fig. 3e–g). Comparing the results presented in Fig. 3 with a representative value for a linear poroelastic solid, following γ_h_ = α(1-2ν)/(1-ν) with the Biot-Willis coefficient α = 1 and Poisson Ratio ν = 0.25, reveals that the mean γ_h_ of the laboratory data is close to the expected value of 0.66 for an isotropic elastic solid^16^. The closely linear correlation between (radial) γ_h_ and the (axial) elastic C_m_ for all samples, seen in Fig. 3e–g, demonstrates that the elastic behavior of the reservoir sandstone is coupled in three dimensions, and hence that the observed 3D constitutive behavior is consistent with linear poroelastic theory^20^. The application of linear poroelastic theory yields a range of Poisson Ratio’s (ν), with an average value around 0.2. The data show a trend that stiffer samples (lower elastic C_m_) display lower Poisson Ratio’s, which is consistent with the behavior expected for isotropic elastic rock material^21,24^. Indeed, it is well-known that the Poisson Ratio ν of sandstone with high compliance is usually low^21,24^. The present dataset therefore is a nice illustration of a rock that exhibits elastic behavior consistent with linear poroelastic theory that is weakly controlled by porosity. Packing and composition do not seem to affect the elastic response much for the present range of samples. An important result of our exercise is that the successful application of poroelasticity is most successful through a careful separation of the elastic-inelastic strain components.

The co-existence of a maximum velocity line, and a clear shear failure line, as presented in Fig. 2, demonstrates that the deformation behavior is brittle in nature, and is at least approaching the critical state line (refer to^22^). Further to the poroelastic response of the Groningen core material, we have observed that up to a factor 0.75 of the total strain was inelastic, and correlated linearly with porosity (Fig. 4a–c), skewness of the particle size distribution (Fig. 4d–f), and quantity of “weak” minerals (Fig. 4g–i). Although the initial crack density was not measured, we have indeed observed microstructurally the presence of intragranular cracks after testing (Fig. 5). From our AE sensing data, we infer that these must have formed at least partly during progressive loading (Fig. 6). The latter confirms, as expected, that the inelastic (permanent) strain is the result of critical grain cracking. DiGiovanni *et al*.^25^ identified a two-stage compaction process in Castlegate sandstone, which was used as analogue for deep sandstone. This process started with a stage of homogeneous grain breakage and re-arrangement, transitioning to localized intense grain breaking. Our data are clearly consistent with the first stage identified by^25^, and hence the deformation measured in our experiments must involve a sequential process where grain breakage and re-arrangement by particle sliding respectively initiate and facilitate displacement and bulk deformation. Future efforts could include quantitative microstructural analysis, notably crack mapping to identify the increase in crack density. Furthermore, the exact role of remaining connate water in sub-critical cracking mechanisms such as stress corrosion cracking^26^ is not clear, as well as potential consequent effects such as creep^27^, which would be independent of the composition of the applied pore fluid. Acid-leaching only resulted in 20% more inelastic axial strain, and no change in γ_h_, which shows that a) only inelastic C_m_ is affected, and b) that the bulk inelastic strain response of our samples is resulting from packing effects and intragranular cracking, as opposed to cement failure. It is therefore important to consider what packing structure and rock phases can control breakage and sliding, i.e. to what extent local strength and mineral friction contribute.

Packing, and the role of particle size distributions in that, is of importance to applications in material science^28^, pharmaceutical technology^29^ and agriculture^30^. In sedimentary geology, a significant number of studies have demonstrated relationships in particle size characteristics for fluvial-aeolian facies^31–33^, similar to the facies considered here in the Groningen field. These studies show that the kurtosis and skewness can often be taken as a proxy for differentiating between beach, dune, and aeolian flat deposits^32^, and beyond this, to e.g. identify relationships between such environments and flora^34^. Considering now a representative distribution for the reservoir rock sampled here reveals that the fine-skewed and very leptokurtic nature found is consistent with aeolian flat deposits. Indeed, the present research well was drilled in the Northern part of the field, where aeolian (dune and flat) deposits prevail over fluvial deposits, in particular in the upper part of the reservoir. By contrast, the Permian located in the Southern area of the Groningen field is characterized by alluvial fan systems that increasingly interact with the aeolian system towards the North^35^.

Particle size distribution properties have a number of known effects on strength, and mechanical behavior in general. The present samples display grain sizes well above the plastic limit of grains (around 1 µm), which suggests that brittle cracking dominates under the present conditions. Since rock constitutive behavior in the brittle field is concerned with the distribution of forces from grain contact to grain contact, the fundamentally most important factor is the coordination number (c.n.). In a pack of grains with similar size, the c.n. is typically fixed, and hence forces can be calculated locally e.g. by combining Hertzian contact mechanics^36^ and linear elastic fracture mechanics^37^, upscaled e.g. by assuming a simple cubic or hexagonal structure^38^. The c.n. has several effects, namely on the bulk strength by packing quality, and by mixing non-identical grains (refer to^39^ for a summary). The latter grain size distribution effect reduces porosity, and increases the c.n., which in turn reduces local stress, and increases bulk strength. In our study, the samples with increasingly fine-skewed particle size distributions display decreasing inelastic C_m_ (Fig. 4), from which we infer that fines in the present aggregates have a stabilizing effect on the packing structure, promoting elasticity. Following from the observations reported by^32^, namely the relationship between skewness, kurtosis and the depositional environment, we here postulate that a relationship could exist between depositional environment and total strain resulting from reservoir depletion. Although insufficient subsurface and rock-specific information exists at field scale, this idea is consistent with the fact that the center of the subsidence bowl is located in the Northern part of the Groningen field. This topic remains subject of further study.

The rocks tested here do exhibit the accumulation of inelastic deformation, or partial bulk failure, during loading, which cannot only be explained by c.n. effects, and must involve distributed material strength in the pack, like others have assumed a Weibull distribution^38^. Strength distribution is the second-most important factor, which in our case is clearly related to composition for samples with a porosity greater than 0.19. Compositional heterogeneity can lead to bulk strength differences. From the extrapolation of the linear fits in Fig. 4g–i, it is clear that in the absence of weak minerals the samples exhibit zero inelastic strain, as such reducing the bulk strain response to a poroelastic problem (refer to origin of plots in Fig. 4g–i). Combining this with the microstructural observations presented in Fig. 5, shows that K-feldspar is frequently in a leached state, and is progressively weakening the rock framework. Although observed mouldic porosity amounts to less than 1.3%, the diametric size of these pores is typically around the mean grain size, which can affect the local stress distributions, and intensify local stress by grain-scale stress-arching. Leaching has also been observed in proximate fields in the North Sea region, where K-feldspar is often replaced by kaolinite^40,41^. Despite the absence of a clear statistically significant regression, we observed also that increasing kaolinite results in weakening. Because the frictional strength of kaolinite is higher than chlorite^42–44^, the weakening effect here is clearly not controlled by its frictional properties but instead by its role in the K-feldspar leaching process, i.e. it rules out the fact that the magnitude of strain is controlled by the frictional properties that resist grain re-arrangement. In summary, the clear consistency between the effect of strong versus weak mineral contrast, and the slight weakening effect of kaolinite versus chlorite in samples with porosity greater than 0.19, again shows that critical cracking controls compaction, in which not only packing, but also K-feldspar leaching plays a crucial role.

K-feldspar content increases towards the top of the reservoir formation, resulting from a change in sedimentary provenance. However, the replacement process (of the K-feldspar by kaolinite) does not appear to be depth-dependent, which suggests the absence of a relationship between the kaolinite and the presence of the gas-water contact, or any diagenetic process^45^. This leads to the general idea that the top part of the gas reservoir, in the Northern part of the area is most susceptible to reservoir compaction, in particular when highly porous, and can both be understood from considerations related to depositional environment.

## Methods

### Sample origin and properties

In July 2015, core material of Permian age was extracted from the near-vertical well ZRP-3 in the Groningen Field, The Netherlands. Obtaining such a core from a field close to depletion poses a number of challenges to drilling engineering, notably related to the mud balance and rock integrity. These were successfully overcome, and the company was able to acquire a total ~200 m of core material from various depth intervals. The intervals cored had a diameter of ~3.5 inch, and included sections of the caprock, reservoir, and underburden rock, preserved in pieces of 0.9 m each. Computerized X-ray Tomography (CT) scans were made of each piece to assist sample selection, using a medical CT scanner with a limited resolution of ~1 mm^3^∙voxel^−1^. Samples were selected from the reservoir section, at a depth between 3530 m and 3667 m, and at irregularly spaced intervals to systematically vary porosity, and sample morphology. To avoid chemical interaction between the pore fluid and the sandstone material during testing, the exact composition of the *in-situ* brine was determined using several ml of pore fluid extracted from the core material by centrifugation. Based on the measured composition, synthetic brine was prepared from NaCl (197.71 g·l^−1^), KCl (4.44 g·l^−1^), CaCl_2_·2H_2_O (147.84 g·l^−1^), MgCl_2_·6H_2_O (25.10 g·l^−1^), and SrCl_2_.6H_2_O (5.20 g·l^−1^) as pore fluid for geomechanical testing, with a composition similar to the field. Since brine salinity is known to have a potentially strong effect on crack propagation processes in sandstone, in particular in the presence of clay^46^, control experiments were conducted using Blandol oil as a pore fluid. From each selected interval (identified with a number, e.g. “02”), three to five plugs measuring 1.0 and 1.5 inch in diameter were drilled parallel to the core axis (identified with “A”, “B”, etc.) having a length of 2.0 inch and 3.0 inch respectively using either synthetic brine or Blandol oil as a cooling fluid. The same fluid was used for storage. The center skeletal core piece was used for conducting Laser particle size analysis. Size distributions were mostly unimodal, occasionally bimodal, and ranged from between 0.5 µm and 2200 µm. Parameterization of the distributions in terms of kurtosis, skewness, mean, and sorting, was performed using the GRADISTAT tool^47^ by applying the geometric method of moments. One of the plugs taken from each numbered layer was used to determine the bulk and solid phase volumes by immersion methods (Hg, Chloroform), from which the porosity fraction was computed under unstressed (ambient) conditions. Moreover, at a constant confining pressure of 2.75 MPa (400 psi), the permeability to N_2_ was determined using the same plugs. X-ray Diffraction (XRD) analysis was performed using bulk rock material, as well as a sieved, clay-sized fraction. The bulk rock analyses showed that the samples contained between a fraction 0.65 and 0.89 of quartz, and up to 0.14 of clay minerals.

### Mechanical testing to determine constitutive response to depletion

**Figure 7:**
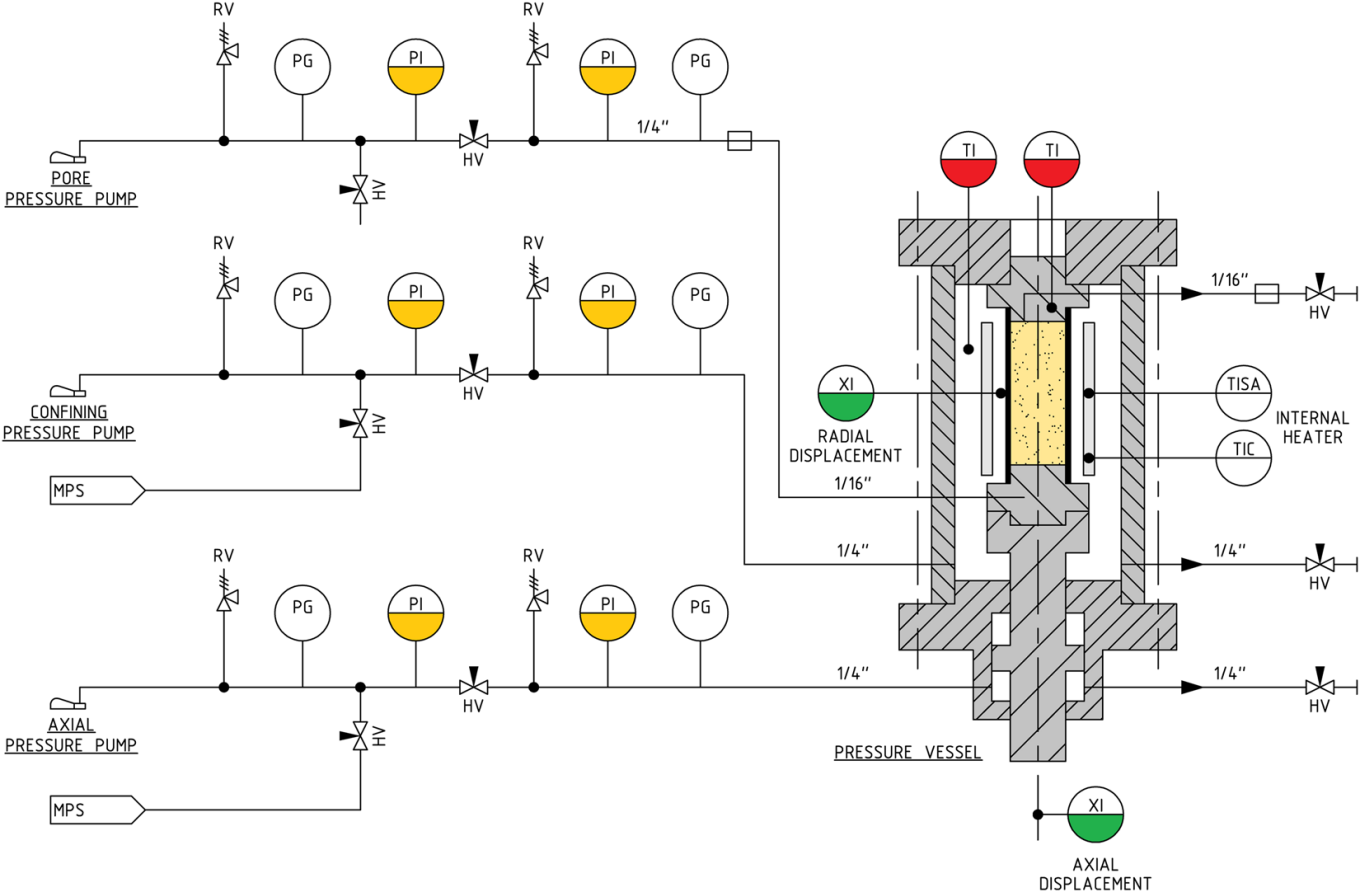
Schematic drawing of a set-up used to perform pore pressure depletion testing using 1′′ diameter samples. The vessels used for testing 1.5′′ diameter samples are similar in design, with the only difference that the axial loading piston is sized to 1.5′′ diameter. The pressure vessel is capable of heating up to 120 °C. The axial pressure (S_ax_), confining pressure (S_rad_) and pore pressures (P_p_), can be applied up to 110 MPa. Abbreviations used are RV = Relief Valve, HV = Hand Valve, MPS = Mobile Pressurization System, PG = Pressure Gauge, PI = Pressure Indicator, TI = Temperature Indicator, XI = Displacement Indicator, TISA = Temperature Indicator Switch Alarm, TIC = Temperature Indicator Controller.

We execute long-term (slow loading) pore pressure depletion tests to accurately determine the mechanical response of the reservoir rock to gas production. The experimental protocols used here are closely consistent with the ISRM Suggested Method for uniaxial-strain testing^9^. Eight independently operated triaxial pressure vessels were used at relevant *in-situ* pressure and temperature conditions (Fig. 7). These systems can hold samples measuring 25.4 mm in diameter and allow testing under various boundary conditions applicable to advancing production from the reservoir, including uniaxial-strain loading (also known as oedometric compaction). Two additional, but similar, triaxial systems can hold samples measuring 38.1 mm in diameter. Enclosed in a 2.5 mm-thick Viton® sleeve, each sample can be mounted onto titanium end caps. After closing the vessel, the system is pressurized using confining oil, and axially by applying oil pressure. The hydraulic pump systems are controlled to within 0.1 MPa by a custom analogue servo-controller, which is limited to 110 MPa. The pressure is measured using a 100 MPa pressure transducer. Pore fluid pressure can be applied to the in- and outlet of the sample, also up to 110 MPa, using either brine or oil, at pore pressure P_p_. Axial displacement is continuously measured, using in each system a Linear Variable Differential Transformer (LVDT) located under the pressure vessel, and connected to an externally positioned Invar frame that translates the relative piston displacement to the sensor (Fig. 7). Radial strain, which represents the strain related to the diametric change of the sample during the test, is measured using strain-gauge assembly in direct contact with the sample. The sensors are all calibrated periodically, which is used to correct for apparatus distortion effects in the measurement and control system during operation. The experimental protocols used in this study are all implemented in a fully automated, pc-controlled manner. Temperature was measured inside the pressure vessel, and pressure lines, using PT100 elements. During several PPD tests experiments, Acoustic Emissions (AE) were recorded using piezoelectric sensors in the frequency range of 100 kHz-1MHz, which were embedded in the axial load piston, and coupled to an IMaGE Cecchi data acquisition unit set at a threshold of 20% of the 2 V full-scale voltage range. Finally, Triaxial Compressive Strength (TCS) tests were performed to determine the overall strength of the sandstone material, taking samples with end-member porosities of 0.13–0.14 and 0.24–0.28. After these tests, the samples were inspected to identify whether the samples fail by developing a distinct shear plane, or by cataclastic flow. The TCS tests were performed in conjunction with ultrasonic p-wave travel time measurements, to identify the transition from compaction-dominated velocity increase, to damage-related velocity decrease.

Using our fully automated triaxial systems, we are able to program a complete test and thereby enabling data comparison later. Each PPD experiment starts by loading the sample to the adopted *in-situ* stress and pore pressure conditions applying stress and pressure (loading rate 1.0 MPa∙hr^−1^) such that isostatic loading, axial loading, and constant net stress loading were such that basic (tangent) elastic constants could be calculated. After a short settling time, the sample was heated to the test temperature and then allowed to settle for mechanical and thermal equilibration for approximately 48 hours. The following versions of the PPD protocol were executed:

TSPPD – Triaxial Stress PPD protocol, whereby the depletion is executed with a fixed γ_h_ of 0.5 or 0.9

UPPD/M – Uniaxial-strain PPD, a multi-cycle protocol used as the standard

UPPD/MC - Uniaxial-strain PPD, a multi-cycle protocol starting at current reservoir pressure

UPPD/ML - Uniaxial-strain PPD, a multi-cycle protocol using acid-leached samples.

UPPD/O - Uniaxial-strain PPD, a one-step protocol

UPPD/OPres - Uniaxial-strain PPD, a one-step protocol starting at current reservoir pressure

For the standard UPPD/M protocol, depletion (and inflation) is carried out in steps of 2.5 MPa∙hr^−1^ pore pressure change under actively controlled uniaxial-strain boundary conditions, while the stress and strain response is actively monitored. The UPPD/ML was executed using two pairs of samples, of which one of the samples in each pair was leached for 24 h using a 50%/50% mixture of synthetic brine, and 10% HCl solution, to investigate the role of cement in compaction.

Stress-strain data were parameterized by applying a linear fit to each loading interval, using poroelastic theory. For small strains, there is no a priori reason to assume that the elastic and inelastic strains are dependent, hence the inelastic deformation component for each depletion step can be computed by subtracting either the strain, or uniaxial-strain compressibility (“C_m_”, strain per unit pore pressure change), of the each third unloading cycle (discussed in Section 2.1) from the first loading step. Here, we have chosen to take the difference in compressibility between total C_m_ obtained during loading, and the elastic C_m_ obtained during unloading, that then equals the inelastic C_m_, which is used for interpreting the constitutive behavior and its relation to the material properties mentioned in 4.1. The other main constitutive response is the tangent change in radial stress as a function of change in pore pressure, parameterized as the horizontal depletion path constant γ_h_^16^ for each pore pressure step. Univariate and multivariate analyses were conducted using the measured response and explanatory variables. Moreover, selective screening of univariate dependencies was performed where appropriate. Data are provided as supplementary documentation, or can be requested by contacting the first author of the present paper.

## Electronic supplementary material

Dataset 1

## References

[1] Thienen-Visser Kv, Breunese JN (2015). Induced seismicity of the Groningen gas field: History and recent developments. The Leading Edge.

[2] Foulger, G. R., Wilson, M., Gluyas, J., Julian, B. R. & Davies, R. Global review of human-induced earthquakes. *Earth-Science Reviews*, 10.1016/j.earscirev.2017.07.008 (2017).

[3] Geertsma J (1973). Land subsidence above compacting oil and gas reservoirs. Journal of Petroleum Technology.

[4] Eshelby JD (1957). The Determination of the Elastic Field of an Ellipsoidal Inclusion, and Related Problems. Proceedings of the Royal Society A.

[5] Brantut N, Heap MJ, Meredith PG, Baud P (2013). Time-dependent cracking and brittle creep in crustal rocks: A review. Journal of Structural Geology.

[6] Atkinson BK (1984). Subcritical crack growth in geological materials. J. Geophys. Res..

[7] Croizé D, Renard F, Gratier JP (2013). In Advances in Geophysics.

[8] Schutjens PMTM (1991). Experimental compaction of quartz sand at low effective stress and temperature conditions. Journal of the Geological Society.

[9] Dudley, J. W. *et al*. ISRM Suggested Method for Uniaxial-Strain Compressibility Testing for Reservoir Geomechanics. *Rock Mechanics and Rock Engineering*, 1–26, 10.1007/s00603-016-1055-4 (2016).

[10] Hettema, M. H. H., Schutjens, P. M. T. M., Verboom, B. J. M. & Gussinklo, H. J. Production-Induced Compaction of a Sandstone Reservoir: The Strong Influence of Stress Path. 10.2118/65410-PA (2000).

[11] de Waal, J. A., van Thienen-Visser, K. & Pruiksma, J. P. Rate Type Isotach Compaction of Consolidated Sandstone. *49th US Rock Mechanics/Geomechanics Symposium* (2015).

[12] Schutjens, P. M. T. M. *et al*. Compaction-Induced Porosity/Permeability Reduction in Sandstone Reservoirs: Data and Model for Elasticity-Dominated Deformation. 10.2118/88441-PA (2004).

[13] Schutjens, P. M. T. M., de Ruig, H., van Muster, J. G., Sayers, C. M. & Whitworth, J. L. Production-Induced Compaction of the Brent Field: An Experimental Approach. 10.2118/28096-PA (1996).

[14] Roholl, J. A., Van Thienen-Visser, K. & Breunese, J. N. Translating Laboratory Compaction Test Results to Field Scale. *50th US Rock Mechanics/Geomechanics Symposium* (2016).

[15] Hol, S., Mossop, A. P., van der Linden, A. J., Zuiderwijk, P. M. M. & Makurat, A. H. Long-term compaction behavior of Permian sandstones - An investigation into the mechanisms of subsidence in the Dutch Wadden Sea. *49th US Rock Mechanics/Geomechanics Symposium* (2015).

[16] Zoback, M. D. *Reservoir Geomechanics*. (Cambridge University Press, 2010).

[17] Wong T-f, Baud P (2012). The brittle-ductile transition in porous rock: A review. Journal of Structural Geology.

[18] Baud P, Klein E, Wong T-f (2004). Compaction localization in porous sandstones: spatial evolution of damage and acoustic emission activity. Journal of Structural Geology.

[19] Chester JS, Lenz SC, Chester FM, Lang RA (2004). Mechanisms of compaction of quartz sand at diagenetic conditions. Earth and Planetary Science Letters.

[20] Wang, H. F. *Theory of linear poroelasticity with applications to geomechanics and hydrogeology*. (Princeton University Press, 2000).

[21] Zimmerman, R. W. *Compressibility of Sandstones*. (Elsevier, 1991).

[22] Rutter EH, Glover CT (2012). The deformation of porous sandstones; are Byerlee friction and the critical state line equivalent?. Journal of Structural Geology.

[23] Lavrov A (2003). The Kaiser effect in rocks: principles and stress estimation techniques. International Journal of Rock Mechanics and Mining Sciences.

[24] Jaeger, J. C., Cook, N. G. W. & Zimmerman, R. W. *Fundamentals of Rock Mechanics*. 4 edn, (Blackwell Publishing, 2007).

[25] DiGiovanni AA, Fredrich JT, Holcomb DJ, Olsson WA (2007). Microscale damage evolution in compacting sandstone. Geological Society, London, Special Publications.

[26] Atkinson BK (1982). Subcritical crack propagation in rocks: theory, experimental results and applications. Journal of Structural Geology.

[27] Heap MJ, Brantut N, Baud P, Meredith PG (2015). Time-dependent compaction band formation in sandstone. Journal of Geophysical Research: Solid Earth.

[28] Bjørk R, Tikare V, Frandsen HL, Pryds N (2013). The Effect of Particle Size Distributions on the Microstructural Evolution During Sintering. Journal of the American Ceramic Society.

[29] Shekunov BY, Chattopadhyay P, Tong HHY, Chow AHL (2007). Particle size analysis in pharmaceutics: Principles, methods and applications. Pharmaceutical Research.

[30] Leonardi C, Shinners KJ, Armentano LE (2005). Effect of different dietary geometric mean particle length and particle size distribution of oat silage on feeding behavior and productive performance of dairy cattle. Journal of Dairy Science.

[31] Martins LR (1965). Significance of skewness and kurtosis in environmental interpretation. Journal of Sedimentary Research.

[32] Mason CC, Folk RL (1958). Differentiation of beach, dune, and aeolian flat environments by size analysis, Mustang Island, Texas. Journal of Sedimentary Research.

[33] Folk RL, Ward WC (1957). Brazos River bar; a study in the significance of grain size parameters. Journal of Sedimentary Research.

[34] González Loyarte MM (2003). Relationship among grain-size, plant communities, and fluvial and eolian processes in a piedmont of the central Andes in Argentina. Ecologia Austral.

[35] Doornebal, H., Stevenson, A., Engineers, E. A. O. G. A. & Survey, B. G. *Petroleum geological atlas of the southern permian basin area*. (European Association of Geoscientists & Engineers (EAGE), 2010).

[36] Johnson, K. L. *Contact mechanics*. 452 (Cambridge University Press, 1985).

[37] Lawn, B. R. *Fracture of brittle solids*. 2nd edn, (Cambridge University Press, 1993).

[38] Brzesowsky, R. H., Spiers, C. J., Peach, C. J. & Hangx, S. J. T. Time-independent compaction behavior of quartz sands. *Journal of Geophysical Research: Solid Earth* **11**9, 10.1002/2013JB010444 (2014).

[39] Mavko, G., Mukerji, T. & Dvorkin, J. *The Rock Physics Handbook - Tools for Seismic Analysis of Porous Media*. 511 (Cambridge University Press, 2009).

[40] Lanson B (2002). Authigenic kaolin and illitic minerals during burial diagenesis of sandstones: a review. Clay Minerals.

[41] Bjorkum PA, Gjelsvik N (1988). An isochemical model for formation of authigenic kaolinite, K-feldspar and illite in sediments. Journal of Sedimentary Research.

[42] Behnsen J, Faulkner DR (2012). The effect of mineralogy and effective normal stress on frictional strength of sheet silicates. Journal of Structural Geology.

[43] Moore DE, Lockner DA (2004). Crystallographic controls on the frictional behavior of dry and water-saturated sheet structure minerals. J. Geophys. Res..

[44] Mitchell, J. K. & Soga, K. *Fundamentals of Soil Behavior* (*3rd Edition*). (John Wiley & Sons, 2005).

[45] Veeningen, R. & Könitzer, S. Petrographic study of well Zeerijp-3A (ZRP-3A). (Panterra Geoconsultants, 2016).

[46] Nara Y (2014). Influences of electrolyte concentration on subcritical crack growth in sandstone in water. Engineering Geology.

[47] Blott SJ, Pye K (2001). GRADISTAT: a grain size distribution and statistics package for the analysis of unconsolidated sediments. Earth Surface Processes and Landforms.

